# A Color-Coded Tape for Uterine Height Measurement: A Tool to Identify Preterm Pregnancies in Low Resource Settings

**DOI:** 10.1371/journal.pone.0117134

**Published:** 2015-03-30

**Authors:** Fernando Althabe, Mabel Berrueta, Jennifer Hemingway-Foday, Agustina Mazzoni, Carolina Astoul Bonorino, Andrea Gowdak, Luz Gibbons, M. B. Bellad, M. C. Metgud, Shivaprasad Goudar, Bhalchandra S. Kodkany, Richard J. Derman, Sarah Saleem, Samina Iqbal, Syed Hasan Ala, Robert L. Goldenberg, Elwyn Chomba, Albert Manasyan, Melody Chiwila, Edna Imenda, Florence Mbewe, Antoinette Tshefu, Victor Lokomba, Carl L. Bose, Janet Moore, Sreelatha Meleth, Elizabeth M. McClure, Marion Koso-Thomas, Pierre Buekens, José M. Belizán

**Affiliations:** 1 Institute for Clinical Effectiveness and Health Policy, Buenos Aires, Argentina; 2 RTI International; Durham, North Carolina, United States of America; 3 KLE University’s Jawaharlal Nehru Medical College, Belgaum, Karnataka, India; 4 Christiana Care, Newark, Delaware, United States of America; 5 Department of Community Health Sciences, Aga Khan University, Karachi Pakistan; 6 Department of Obstetrics, Sobhraj Maternity Hospital, Karachi, Pakistan; 7 Department of Obstetrics, Sindh Government Qatar Hospital, Karachi Pakistan; 8 Department of Obstetrics and Gynecology, Columbia University, New York, New York, United States of America; 9 University Teaching Hospital, Lusaka, Zambia; 10 Centre for Infectious Disease Research in Zambia, Lusaka, Zambia; 11 Kinshasa School of Public Health, Kinshasa, Democratic Republic of Congo; 12 University of North Carolina, Chapel Hill, North Carolina, United States of America; 13 Eunice Kennedy Shriver NICHD, Bethesda, Maryland, United States of America; 14 School of Public Health and Tropical Medicine, Tulane University, Louisiana, United States of America; Seattle Childrens Hospital, UNITED STATES

## Abstract

**Introduction:**

Neonatal mortality associated with preterm birth can be reduced with antenatal corticosteroids (ACS), yet <10% of eligible pregnant women in low-middle income countries. The inability to accurately determine gestational age (GA) leads to under-identification of high-risk women who could receive ACS or other interventions. To facilitate better identification in low-resource settings, we developed a color-coded tape for uterine height (UH) measurement and estimated its accuracy identifying preterm pregnancies.

**Methods:**

We designed a series of colored-coded tapes with segments corresponding to UH measurements for 20–23.6 weeks, 24.0–35.6 weeks, and >36.0 weeks GA. In phase 1, UH measurements were collected prospectively in the Democratic Republic of Congo, India and Pakistan, using distinct tapes to address variation across regions and ethnicities. In phase 2, we tested accuracy in 250 pregnant women with known GA from early ultrasound enrolled at prenatal clinics in Argentina, India, Pakistan and Zambia. Providers masked to the ultrasound GA measured UH. Receiver operating characteristics (ROC) analysis was conducted.

**Results:**

1,029 pregnant women were enrolled. In all countries the tapes were most effective identifying pregnancies between 20.0–35.6 weeks, compared to the other GAs. The ROC areas under the curves and 95% confidence intervals were: Argentina 0.69 (0.63, 0.74); Zambia 0.72 (0.66, 0.78), India 0.84 (0.80, 0.89), and Pakistan 0.83 (0.78, 0.87). The sensitivity and specificity (and 95% confidence intervals) for identifying pregnancies between 20.0–35.6 weeks, respectively, were: Argentina 87% (82%–92%) and 51% (42%–61%); Zambia 91% (86%–95%) and 50% (40%–60%); India 78% (71%–85%) and 89% (83%–94%); Pakistan 63% (55%–70%) and 94% (89%–99%).

**Conclusions:**

We observed moderate-good accuracy identifying pregnancies ≤35.6 weeks gestation, with potential usefulness at the community level in low-middle income countries to facilitate the preterm identification and interventions to reduce preterm neonatal mortality. Further research is needed to validate these findings on a population basis.

## Introduction

Preterm birth is the leading cause of child mortality and interventions are available to reduce this mortality [1–3]. Among the most effective perinatal intervention to reduce neonatal mortality associated with preterm birth is the administration of antenatal corticosteroids (ACS) to pregnant women at high risk of preterm birth. However, the use of ACS and other interventions are limited in low and middle-income countries (LMIC) [2–7] because it is difficult to accurately determine gestational age (GA) in these settings, where ultrasound assessment is often unavailable [8, 9]. Estimating GA by last menstrual period (LMP) date and early ultrasound are considered the most accurate methods in settings where women commonly record their LMP and where ultrasound assessment is the norm. In LMIC, neither of these practices is common [10–13]. Many factors contribute to the inability to determine GA in LMIC, including lack of the dates of last menstrual periods (LMP) [10,11], high rates of care provided by traditional birth attendants (TBAs) who cannot calculate GA [12] and limited access to ultrasound devices [13]. Furthermore, for women with unknown GA who receive antenatal care at the community level, there are no simple, accurate methods for traditional and skilled birth attendants to identify women in the gestational ages range at-risk for preterm birth (e.g., 24 to 36 weeks GA), thus inhibiting the provision of antenatal corticosteroids and early referral. Hence, whether at the community or primary health care level, public health strategies would benefit from an accurate, simple method that improves the capacity of birth attendants to identify GA for women without a reliable estimate of GA. To address these concerns, we developed a uterine height (UH) measurement tool to assess the GA for health providers lacking literacy skills and tools to accurately identify women at risk of preterm delivery in low resource settings.

UH measurement is widely used for screening of intra-uterine growth restriction in LMIC [14–16]. In these settings, procedures have been designed so that even untrained, illiterate health providers can implement the measurements. For example, to facilitate UH measurement of fetal growth by indigenous midwives in Guatemala, Villar and colleagues designed a measuring tape with colored zones of UH measurements corresponding to small-for-gestational-age babies [16]. Other groups evaluating UH as a proxy for GA have shown that it is an accurate method to determine GA when LMP is unknown [17–19]. However, these studies evaluated measuring tapes that required some level of literacy, which is often unrealistic for TBAs [12]. To date, no basic UH measuring tape to estimate GA has been designed for use in settings where providers lack literacy skills.

To facilitate birth attendants’ identification of pregnant women at high risk for preterm birth in community-based settings with limited provider literacy, we designed a color-coded measuring tape to measure UH and tested its accuracy to identify women who were likely to deliver preterm. The study was conducted within the *Eunice Kennedy Shriver* National Institute of Child Health and Human Development’s (NICHD) Global Network for Women’s and Children’s (Global Network) [20], and as part of its Antenatal Corticosteroids Trial (ACT)[8].

### The Global Network Antenatal Corticosteroids Trial

The ACT cluster randomized controlled trial was conducted in more than 100 communities and facilities in Argentina, Guatemala, India, Kenya, Pakistan, and Zambia under the NICHD’s Global Network [8]. Its main objective was to evaluate a complex intervention that facilitates both the identification of women at high risk for preterm birth and the administration of antenatal corticosteroids, with the aim of reducing neonatal mortality. The goal of developing and evaluating the color-coded measuring tape was to provide a tool that would facilitate identification of women eligible for this study, which included women at high risk for preterm birth between 24.0 and 36.0 weeks.

## Materials and Methods

### Development of the color-coded measuring tape

The first phase of the study was conducted between June 2007 and December 2009, with 2,434 women enrolled in participating sites in the Democratic Republic of the Congo [DRC] (Kinshasa), India (Karnataka), and Pakistan (Karachi). All women who presented for antenatal care at participating hospitals were screened for eligibility. Informed consent was requested of women with a GA between 24 and 36 weeks, according to an early ultrasound (prior to 20 weeks GA), with a live fetus with no identified malformations; and without pregnancy or medical complications. Two independent operators, generally physicians or nurses, used a non-stretchable measuring tape to record the UH in centimeters. At each study site, the operators, one of whom was unaware of the ultrasound GA, measured 30–50 women for each week of GA. We evaluated agreement between the two operators using Bland-Altman method [21]. As there was an unbiased high correlation of the two measurements (S1 Fig) with a 2–3 cm difference in 95% of the measurements, each woman’s mean UH was calculated from the two measurements taken. From these mean values, we then calculated mean and standard deviation, Percentile 10 (P10), P50, P90 UH measurements (in centimeters) corresponding to each week between 24 and 36 weeks for each study site. These measurements for each week of GA by site are shown in S1 Table.

Measurements of UH from these study sites were used to develop a series of color-coded measuring tapes to categorize pregnancies. Each tape has three colored segments: a yellow zone corresponding to a gestational age <24.0 weeks; a red zone corresponding to 24.0 to 36.0 weeks; and a green zone corresponding to >36.0 weeks. Combinations of UH measurements corresponding to the P10, P50, and P90 for pregnancies at the 24.0th and 36.0th week of GA were used as lower and upper limits of the color-coded zones on the measuring tapes (S1 Table). Each tape represented a different cut-off point for the diagnostic test. For example, the range of each category on the 50–50 tape was defined by the P50 for the 24^th^ and 36^th^ week of gestation. The range of each category on the 10–90 tape was defined by the P10 at 24 weeks and the P90 at 36 weeks.

**Fig 1:**
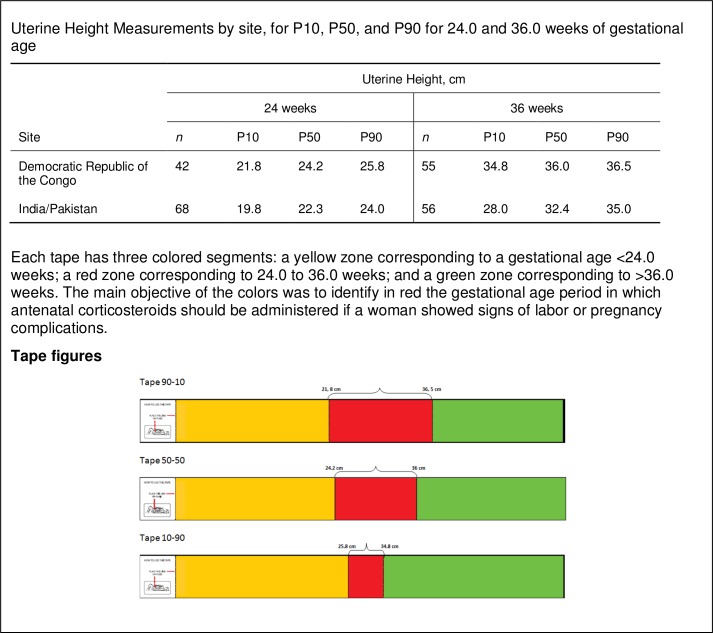
Design of the color-coded tapes.

We developed two sets of tri-color-coded tapes. The first set was based on the DRC measurements, but it was not logistically feasible to test the accuracy of the tapes in DRC. Instead, this tape was evaluated in Africa and Latin America, as secondary data obtained from the study sites of mean birth weight, fundal height, and anthropometric measures from Zambia, Argentina and Guatemala indicated that the DRC measurements could be appropriate these settings. A second tape was based on the combined data from India and Pakistan and was evaluated in those two countries. The measurements from India and Pakistan sites were combined, as the anthropometric characteristics of the women, mean birth weight, and measured uterine heights were very similar (data not shown) [14,20]. The tapes, made of a tear-proof, non-stretchable reinforced paper material, were produced centrally by the Global Network Data Coordinating Center (DCC), Research Triangle Institute (RTI). Fig. 1 shows those measurements, characteristics and figures of the color-coded tapes.

### Estimation of the diagnostic accuracy of the color-coded tape to identify pregnancies in the preterm GA range

#### Settings and Participants

After developing the tape, a prospective cross-sectional study was conducted in a subset of the Global Network’s hospitals, within antenatal clinics at the University Teaching Hospital in Zambia; KLES Dr Prabhakar Kore Hospital and Medical Research Centre in Belgaum, India; Sindh Government Qatar Hospital Karachi and Sobhraj Maternity Hospital in Karachi, Pakistan; and Maternidad “Ramón Sardá” in Buenos Aires, Argentina between October 2010 and February 2012. For logistical reasons, the Zambia study site replaced the DRC site during this phase. Women presenting at these sites were screened for eligibility when the study team was available. Informed consent was requested of women with a live singleton fetus, with a GA between 20.0 and 40.6 weeks confirmed by ultrasound scan performed before 20 weeks gestation (the reference standard). To avoid double recruitment, a note about study participation was made in the antenatal clinical record and recruitment card.

We enrolled a target sample of 250 pregnant women per site, with the following GA distribution: 50 women between 20.0 and 23.6 weeks, 100 preterm women between 24.0 and 35.6 weeks (10 between 24.0 and 27.6 weeks, 20 between 28.0 and 31.6 weeks, and 70 between 32.0 and 35.6 weeks), and 100 women between 36.0 and 40.6 weeks. Assignment of gestational age was based on early ultrasound. Stratification of the sampling distribution for women in the preterm GA category was designed to approximate the specific GA distribution expected for preterm births at Global Network sites [20,22]. For each woman, a gestational age range was assigned independently using the color-coded tape.

At each site, data were collected on paper forms specially designed for this study, entered into a data management system by a data entry clerk, and then transmitted through a secure transmission to the DCC where the data were compiled, cleaned and analyzed.

### Ethics Statement

Ethical approval was obtained from the Institutional Review Boards (IRBs) of the Data Coordinating Center, RTI International, Research Triangle Park, North Carolina and the lead institution, Tulane University, New Orleans, Louisiana. Approval was also obtained from local implementing institutions and their U.S. partners: For Argentina, approval was obtained from the IRB at Tulane University, and the Ethical Research Committees (ERCs) at Centro de Educacíon Médica E Investigaciones Clínicas “Norberto Quirno” and the Sardá Hospital, both located in Buenos Aires, Argentina; for the DRC, approval was obtained from the ERC at the Kinshasa School of Public Health, Kinshasa, DRC and the IRB at the University of North Carolina, Chapel Hill, North Carolina; for India, approval was obtained from the Institutional Ethics Committee (IEC) at Jawaharlal Nehru Medical College, Belgaum, India and the IRB of Christiana Care Health Systems, Newark, Delaware; for Pakistan, approval was obtained from the ERC at Aga Khan University, Karachi, Pakistan and the IRB at Drexel University College of Medicine, Philadelphia, Pennsylvania; and for Zambia, approval was obtained from the Research Ethics (RE) School of Medicine at the University Teaching Hospital, Lusaka, Zambia and the IRB at University of Alabama, Birmingham, Alabama. All study participants provided written informed consent. The consent form was provided in the local language and accommodations were made for low-literacy participants by accepting either a signature or thumb print as written confirmation of consent.

### Test methods

#### Procedure.

After consent, one unmasked health provider obtained information regarding the patients’ background, reproductive characteristics, and their ultrasound determined GA. An independent operator (midwives or obstetricians, including medical residents, who provide antenatal care at participating hospitals), who was masked to the patients’ GA, then measured their UH with the tri-colored study tapes and recorded the color zone corresponding to each tape’s measurement of UH on a separate data form. Only a subject study number linked both data forms. Data were recorded on study paper forms, entered on site into a data management system, and transmitted through a secure system to the DCC where the data were compiled, cleaned and analyzed.

#### UH Measurement Technique.

**Fig 2:**
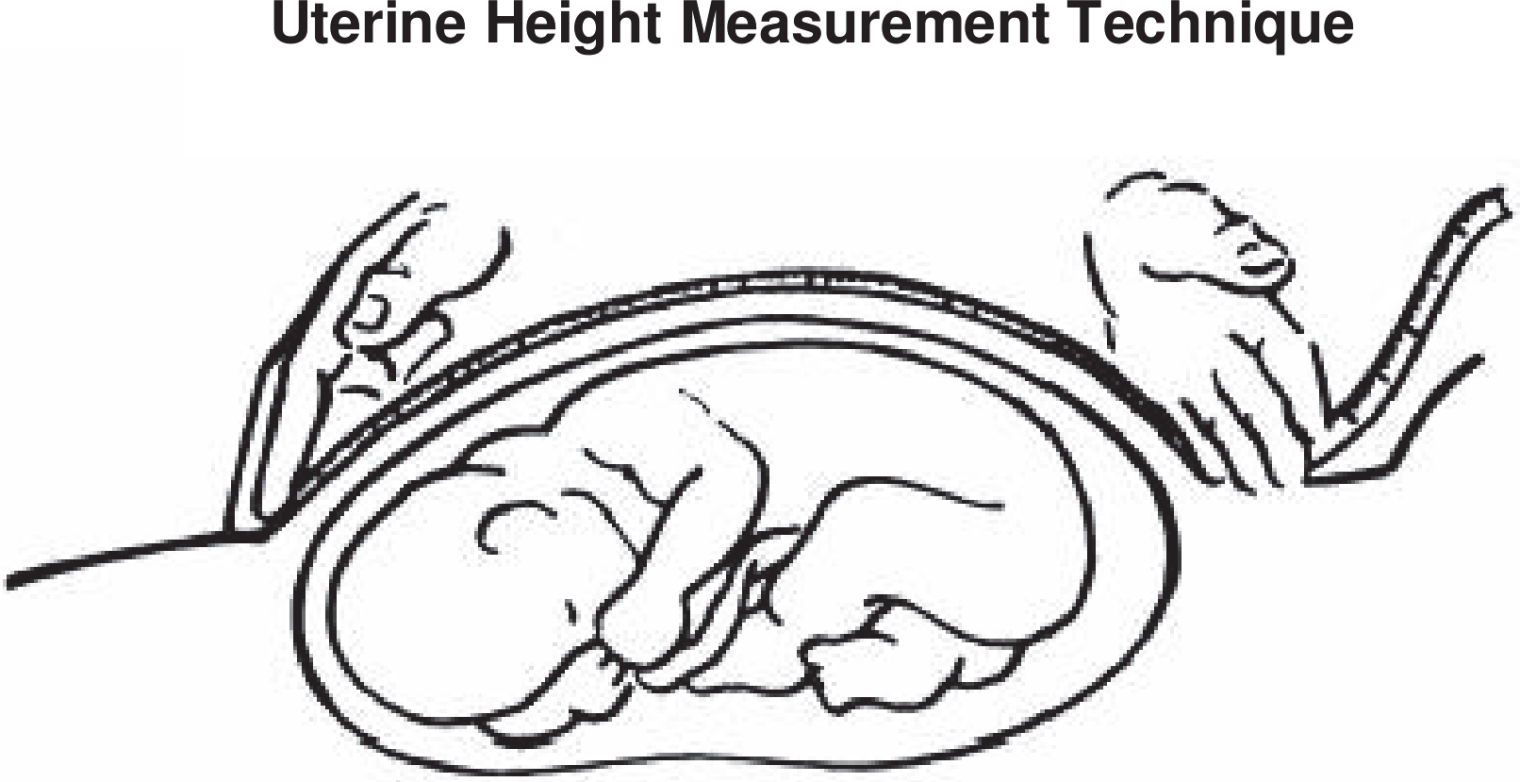
Uterine Height Measurement Technique.

Between 2–4 operators per hospital were trained in all study procedures by viewing a video designed for the study, which included a step-by-step description of the enrollment guidelines, measurement technique, and data collection procedures. The video is available upon request. To obtain the UH measurement, the tape was placed on the maternal abdomen, with the mother in a supine position, with the colored side facing the belly and blinded to the operator. One end of the tape was placed at the upper edge of the pubic bone. Pressing down firmly but gently with the other hand, the operator extended the tape to the center-point of the uterine fundus, holding the tape with the cubital edge of the hand (**Fig. 2.** Adapted from Villar et al., 1979 [16]).

#### Statistical Methods.

For each site, we calculated the sensitivity and specificity [and 95% confidence intervals (CIs)] of the three versions of the colored-coded tapes to identify: a) pregnancies between 24.0 and 35.6 weeks of GA and b) pregnancies between 20.0 and 35.6 weeks of GA. For evaluating the accuracy of the tape in identifying pregnancies from 24.0 to 35.6 weeks, a positive test was a uterine height measurement that fell within the red zone on the tape, and a negative test was a uterine height measurement that fell either within the green or the yellow zones. For evaluating the accuracy of the tape in identifying pregnancies from 20.0 to 35.6 weeks, a positive test was a uterine height measurement that fell within the red or yellow zones and a negative test was a uterine height in the green zone. Receiver Operating Curves (ROC) were plotted with 95% CIs, and the areas under the curves were calculated for each site based on the results of the three tapes [23,24].

We calculated that a sample of 250 pregnant women per site (comprised of 100 women with a GA between 24 and 35.6 weeks) would allow us to assess the sensitivity and specificity of the color-coded measuring tapes to identify preterm pregnancies (i.e., between 24 and 36 weeks) with a 95% CI of 82%–96%, for a 90% sensitivity. Analyses were done with SAS v 9.3 (Cary, NC). Reporting was done following the STARD standards [25].

## Results

### Participants

**Fig 3:**
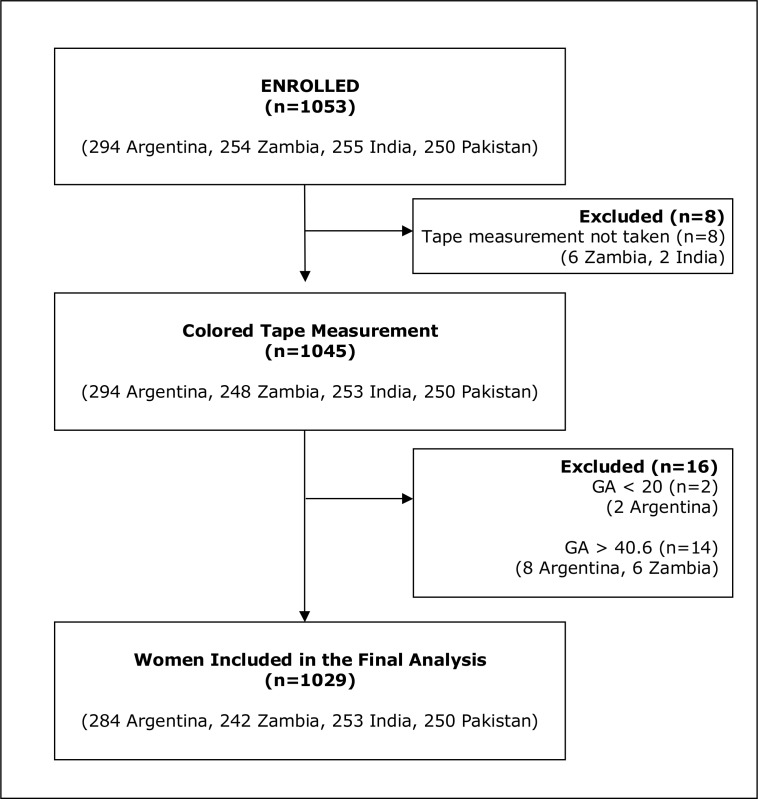
Flow Diagram by Country.

A total of 1,029 eligible women were enrolled in the study (284 in Argentina, 242 in Zambia, 253 in India, and 250 in Pakistan). Fig. 3 shows the study diagram and Table 1 summarizes the characteristics of the women. The GA distribution of enrolled women followed the convenience-sampling plan described above.

**Table 1:** Characteristics of the participating pregnant women.

	Argentina	Zambia	India	Pakistan
Age—Mean (SD)	25.3 (5.7)	29.4 (5.0)	23.0 (3.2)	25.1 (4.2)
Height (cm)—Mean (SD)	160.6 (73.3)	161.6 (6.4)	153.2 (5.2)	155.3 (5.1)
Weight (kg)—Mean (SD)	69.6 (13.0)	78.0 (16.3)	55.0 (9.5)	62.1 (12.2)
Gestational Age (weeks)—N	284	242	253	250
20.0 to 23.6	39 (13.7)	34 (14.0)	46 (18.2)	48 (19.2)
24.0 to 27.6	35 (12.3)	25 (10.3)	14 (5.5)	12 (4.8)
28.0 to 31.6	39 (13.7)	28 (11.6)	24 (9.5)	23 (9.2)
32.0 to 35.6	64 (22.5)	63 (26.0)	55 (21.7)	67 (26.8)
36 to 40.6	107 (37.7)	92 (38.0)	114 (45.1)	100 (40.0)

### Test Results

Table 2 shows, by site and tape design, a cross tabulation of the results of the color-coded tape measurements (yellow, red, and green colored zones) by gestational age compared with the reference standard GA as determined by ultrasound (20.0–23.6, 24.0–35.6, 36.0–40.6 weeks).

**Table 2:** Distribution of tape colored zone measurements compared to the actual gestational age as determined by ultrasound in each site.

	Argentina			Zambia			India			Pakistan		
	20.0–23.6	24.0–35.6	36.0–40.6	20.0–23.6	24.0–35.6	36.0–40.6	20.0–23.6	24.0–35.6	36.0–40.6	20.0–23.6	24.0–35.6	36.0–40.6
Tape “10–90”												
Yellow	0	10	32	0	10	28	0	2	5	0	7	20
Red	29	126	75	6	92	64	5	83	108	36	95	80
Green	10	2	0	28	14	0	41	8	1	12	0	0
Tape “50–50”												
Yellow	0	14	32	0	9	35	0	4	24	0	17	51
Red	23	113	73	4	82	57	1	71	89	15	78	49
Green	16	11	1	30	25	0	45	18	1	33	7	0
Tape “90–10”												
Yellow	0	23	55	0	14	46	0	31	101	1	55	94
Red	15	97	50	1	67	44	0	41	12	5	35	6
Green	24	18	2	33	35	2	46	21	1	42	12	0

### Estimates of accuracy

**Fig 4:**
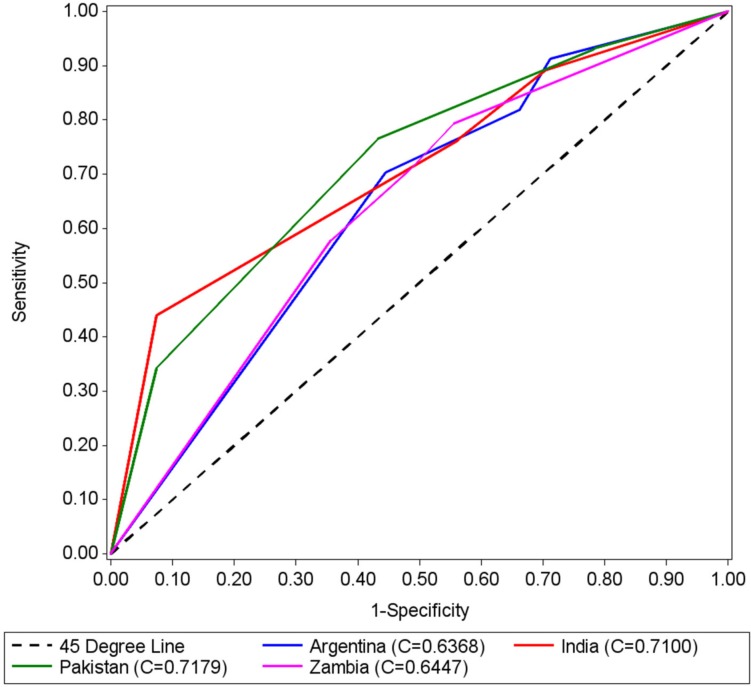
ROC Curves by Country for 24–35.6 weeks of gestational age.

**Fig 5:**
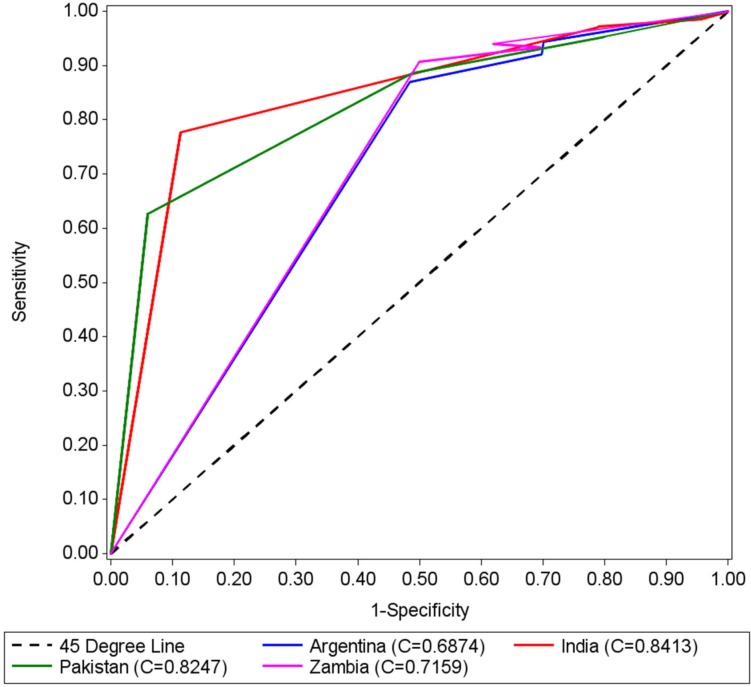
ROC Curves by Country for 20–35.6 weeks of gestational age.

ROC curves were generated by site to depict the accuracy of the tapes in identifying pregnancies with GA between 24.0 to 35.6 weeks and GA between 20.0 to 35.6 weeks (Figs. 4 and 5, respectively). The areas under the curves (AUCs) that tested identification of pregnancies between 24.0 to 35.6 weeks GA, by country, were (with 95% CIs): Argentina 0.64 (0.58, 0.70), Zambia 0.65 (0.58, 0.71), India 0.71 (0.65, 0.78), and Pakistan 0.72 (0.66, 0.78). AUCs that tested identification of pregnancies between 20.0 to 35.6 weeks were: Argentina 0.69 (0.63, 0.74); Zambia 0.72 (0.66, 0.78), India 0.84 (0.80, 0.89), and Pakistan 0.83 (0.78, 0.87). ROC analysis showed that in all countries the tapes were more effective in identifying pregnancies between 20.0 to 35.6 weeks, and that the tape designed using percentiles 90 and 10 (tape 90–10) provided the highest level of accuracy. The curves illustrated that the color-coded tapes performed better in India and Pakistan than in Argentina and Zambia.

The sensitivity and specificity of the three tapes for identifying pregnancies between 20.0 to 35.6 and 24.0 to 35.6 weeks are summarized in Table 3. For the most precise tape—the 90–10 design—the sensitivity and specificity for identifying pregnancies between 20.0 to 35.6 weeks, respectively, were: Argentina 87% (82%–92%) and 51% (42%–61%); Zambia 91% (86%–95%) and 50% (40%–60%); India 78% (71%–85%) and 89% (83%–94%); Pakistan 63% (55%–70%) and 94% (89%–99%).

**Table 3:** Sensitivity and Specificity of the color-coded tape to identify pregnancies with GA between24.0–35.6 weeks and between 20.0–35.6 weeks.

**Sensitivity and Specificity of the color-coded tape to identify pregnancies with GA between24.0–35.6 weeks**				
	Argentina	Zambia	India	Pakistan
Sensitivity				
Tape 10–90	91.3 (86.6, 96.0)	79.3 (71.9, 86.7)	89.2 (83.0, 95.5)	93.1 (88.2, 98.0)
Tape 50–50	81.9 (75.5, 88.3)	70.7 (62.4, 79.0)	76.3 (67.7, 85.0)	76.5 (68.2, 84.7)
Tape 90–10	70.3 (62.7, 77.9)	57.8 (48.8, 66.7)	44.1 (34.0, 54.2)	34.3 (25.1, 43.5)
Specificity				
Tape 10–90	28.8 (21.4, 36.1)	44.4 (35.8, 53.1)	29.4 (22.3, 36.4)	21.6 (15.0, 28.3)
Tape 50–50	33.8 (26.1, 41.5)	51.6 (42.9, 60.3)	43.8 (36.1, 51.4)	56.8 (48.8, 64.7)
Tape 90–10	55.5 (47.4, 63.5)	64.3 (55.9, 72.7)	92.5 (88.4, 96.6)	92.6 (88.3, 96.8)
**Sensitivity and Specificity of the color-coded tape to identify pregnancies with GA between 20.0–35.6 weeks**				
	Argentina	Zambia	India	Pakistan
Sensitivity				
Tape 10–90	94.4 (90.9, 97.8)	93.3 (89.3, 97.3)	98.6 (96.6, 100.5)	95.3 (92.0, 98.7)
Tape 50–50	92.1 (88.1, 96.1)	94.0 (90.2, 97.8)	97.1 (94.3, 99.9)	88.7 (83.6, 93.7)
Tape 90–10	87.0 (82.1, 92.0)	90.7 (86.0, 95.3)	77.7 (70.8, 84.6)	62.7 (54.9, 70.4)
Specificity				
Tape 10–90	29.9 (21.2, 38.6)	30.4 (21.0, 39.8)	4.4 (0.6, 8.1)	20.0 (12.2, 27.8)
Tape 50–50	30.2 (21.4, 38.9)	38.0 (28.1, 48.0)	21.1 (13.6, 28.5)	51.0 (41.2, 60.8)
Tape 90–10	51.4 (41.9, 60.9)	50.0 (39.8, 60.2)	88.6 (82.8, 94.4)	94.0 (89.3, 98.7)

## Discussion

The study showed that the color-coded measuring tapes had moderate to good accuracy in identifying pregnancies < = 35.6 weeks GA. Using the tape 90–10, which produced the most accurate GA estimates, the observed sensitivity in Argentina and Zambia was high (87% and 91% respectively), but specificity was low (51% and 50%). In India and Pakistan, however, sensitivity was moderate (78% and 63% respectively) but specificity was high (89% and 94%). Overall, the ROC analysis suggested that the test performed better in India and Pakistan. The AUCs suggested that if the tape was used to assess which of two pregnant women (one with gestational age < = 35.6 weeks and another between 36.0 to 40.0) had a preterm pregnancy, the test would be correct 83% and 84% of the times in Pakistan and India, and 69% and 72% in Argentina and Zambia, respectively.

The most likely explanation of the different performance between the two groups of countries is that the tapes used in Argentina and Zambia were designed by using UH measurements taken in another country (DRC), whereas the tapes used in India and Pakistan were designed using UH measurements from their own populations. This assumption is also supported by the close similarity in the accuracy observed in India and Pakistan, compared to those in Argentina and Zambia. The tape showed a lower sensitivity to identify pregnancies between 24.0 to 35.6 weeks.

The study has several strengths. For both study phases (developing the tape and evaluating its accuracy), the GA of each pregnant woman was confirmed by an ultrasound prior to 20 weeks of gestation; midwives and nurses received a standardized training and used the same procedures across sites; and all UH measurements were carried out by operators who were masked to the actual gestational age.

However, the study also has limitations. First, the study included women receiving antenatal care at hospitals, with GA assessment and no acute pregnancy complications. It is possible that the UH of pregnant women either without antenatal care, or with unknown GA, or who face emergency situations due to pregnancy complications may be slightly lower than women in the study sample receiving antenatal care. For example, women either with premature rupture of membranes, preeclampsia with intrauterine growth restriction, or in labor, have been found to have lower UH at the same gestational age as women without those complications [26]. Those emergencies are the target situations in which the tape could be useful. Thus it is possible that, when used in routine clinical practice, the evaluated tapes would have higher sensitivity, but lower specificity. Second, by sampling pregnancies < = 35.6 weeks (“cases”) and pregnancies between 36.0 and 40.6 weeks (“referents”), the “prevalence” of women with pregnancies in the preterm GA range was artificially optimized (i.e. 60% of sample women). In this case, variation in prevalence can be a source of different “reader expectations” [24], which may influence the accuracy of the tests by changing the implicit threshold that the health providers have to define a case positive. The expectation of the health providers to detect a preterm pregnancy in this study with a 60% “prevalence” of preterm pregnancies may be different than a real-world setting with a lower prevalence of women with preterm pregnancies.

The color-coded tape was developed as part of a multifaceted intervention that aimed to increase the use of antenatal steroids for women at risk of preterm birth. As part of the intervention, health providers were trained to use the tape at the community level for pregnant women with unknown gestation age that presented with emergency clinical conditions (labor, rupture of membranes, obstetric hemorrhage, or severe hypertension). As an example, in India, assuming a 20% prevalence of preterm pregnancies in women with such conditions, based on the observed accuracy of the tape 90–10, 25% of women would be classified as preterm (16% true preterm and 9% false positives diagnosis); and 75% would be classified as term pregnancies (71% true term and 4% of false negative results).

While our results would ideally be confirmed in a phase III diagnostic study, such a study is unlikely to be feasible because the very communities where it could make the greatest impact are those without access to ultrasound services to validate the results. Thus, without the availability of an ultrasound device to obtain reference GA, testing the accuracy of these tapes is not possible. Only women who are identified as preterm and are effectively referred to a higher level of care with ultrasound services would have an accurate estimate of GA, the assessment of positive predictive and false positive values being the only characteristics possible to be assessed. Clinicians and policymakers should therefore decide whether the results of this phase II study provide enough evidence to support implementation of the color-coded measuring tape in operational conditions. Based on our results, additional color-coded measuring tapes should preferably be developed using tailored UH measurements for gestational age charts in each population. With confirmation of our results under actual conditions, the color-coded measuring tape would be a useful tool in LMIC, to facilitate the assessment of gestational age and the identification of women at risk for preterm birth, and ultimately to increase the appropriate use of antenatal steroids and other interventions to reduce neonatal mortality.

## Supporting Information

S1 Fig Bland-Altman plots showing inter-observer agreement between the fundal height measurements taken by two operators, by site. (DOCX)

S1 Table Fundal height by gestational age by region. (DOCX)
