# Supplementary material for: A Color-Coded Tape for Uterine Height Measurement: A Tool to Identify Preterm Pregnancies in Low Resource Settings
Source: PLoS One. 2015 Mar 30;10(3):e0117134. doi: 10.1371/journal.pone.0117134 (PMC4379082; doi:10.1371/journal.pone.0117134)
Supplement: S1 Fig — (DOCX) [file pone.0117134.s001.docx]

|  |
| --- |

| Figure A: Bland-Altman Plot for 02 DRC |
| --- |

|  |
| --- |

| Figure B: Bland-Altman Plot for 08 Belgaum |
| --- |

|  |
| --- |

| Figure C: Bland-Altman Plot for 09 Pakistan |
| --- |
