# Supplementary material for: A Color-Coded Tape for Uterine Height Measurement: A Tool to Identify Preterm Pregnancies in Low Resource Settings
Source: PLoS One. 2015 Mar 30;10(3):e0117134. doi: 10.1371/journal.pone.0117134 (PMC4379082; doi:10.1371/journal.pone.0117134)
Supplement: S1 Table — (DOCX) [file pone.0117134.s002.docx]

**Africa (DRC)**

**Median Fundal Height**

| GA | N | Median | 10th Pctl | 90th Pctl |
| --- | --- | --- | --- | --- |
| 24 | 49 | 24.3 | 21.8 | 25.8 |
| 25 | 49 | 25.2 | 23.3 | 27.3 |
| 26 | 52 | 25.8 | 23.2 | 28.3 |
| 27 | 50 | 26.4 | 23.5 | 28.7 |
| 28 | 48 | 26.8 | 24.8 | 29.3 |
| 29 | 51 | 28.3 | 25.7 | 29.5 |
| 30 | 52 | 30.0 | 28.0 | 32.4 |
| 31 | 44 | 31.3 | 28.5 | 33.3 |
| 32 | 52 | 31.0 | 29.3 | 33.8 |
| 33 | 46 | 32.4 | 29.3 | 35.3 |
| 34 | 44 | 34.0 | 31.5 | 35.3 |
| 35 | 45 | 34.5 | 31.4 | 35.5 |
| 36 | 89 | 36.0 | 33.3 | 36.5 |

**Asia (India and Pakistan)**

**Median Fundal Height**

| GA | N | Median | 10th Pctl | 90th Pctl |
| --- | --- | --- | --- | --- |
| 24 | 75 | 22.5 | 20.0 | 24.0 |
| 25 | 72 | 23.5 | 20.8 | 26.5 |
| 26 | 89 | 24.0 | 21.0 | 26.0 |
| 27 | 93 | 25.5 | 22.0 | 27.5 |
| 28 | 102 | 26.0 | 22.0 | 28.3 |
| 29 | 92 | 27.0 | 24.0 | 29.5 |
| 30 | 84 | 28.0 | 24.5 | 30.0 |
| 31 | 88 | 29.0 | 26.0 | 31.3 |
| 32 | 110 | 30.0 | 26.6 | 31.5 |
| 33 | 103 | 30.5 | 26.0 | 32.5 |
| 34 | 111 | 31.3 | 28.0 | 33.0 |
| 35 | 108 | 32.5 | 28.0 | 33.8 |
| 36 | 67 | 32.3 | 28.0 | 35.0 |
